# Medical Association of Alabama

**Published:** 1875-05

**Authors:** 


### Our State Societies.

### ALABAMA MEDICAL ASSOCIATION.

MONTGOMERY, ALA., April 13, 1875.

*Editor Atlanta Medical and Surgical Journal:*

The State Medical Association of Alabama was called to order to-day at 12 o'clock, by the President, Dr. J. S. Weatherly, of Montgomery, a full attendance being present; among them many of the most distinguished medical men of the State.

After a feeling prayer by Rev. Dr. Stringfellow, an address of welcome was delivered by Dr. W. C. Jackson, of this city, tendering to the audience present a hearty and cordial welcome, and the Association a harmonious meeting.

On motion, the President's address was postponed until 8 o'clock this evening. The meeting was then declared open for the transaction of business.

On motion, Drs. Griggs and Kendrick, delegates from the Medical Association of Georgia, were invited to seats as delegates from that Association.

The report of the Secretary, Dr. B. H. Riggs, was read and adopted. It was concise and interesting, and closed by saying that many valuable books and periodicals had come into his possession during the year, and thinks the time not far distant when a library will be collected of considerable value. Has written many letters during the year to advance the interest of the Association. Recommended the appointment of delegates to the various State Associations, etc.

The report of the Treasurer, full and elaborate, was then read and approved.

The meeting of the Board of Censors was postponed until to-morrow.

Reports of various medical societies throughout the State were listened to with interest.

Motion prevailed to compliment the Medical Association of South Carolina, now in session at Charleston, by telegraph, with the fraternal regards of this body.

In the absence of the annual orator, Dr. George A. Ketchum, of Mobile, was appointed to address the Association and public, to-morrow evening at 8 o'clock.

Meeting adjourned.

Met at 2½ o'clock P.M.

Dr. Gaines, of Mobile, consumed some time in reading and discussing an interesting paper on Tuberculosis. He drew a comparison between tuberculosis and scrofulosis. He believes scrofulosis to be an inflammatory disease, tuberculosis non-inflammatory. Tuberculosis is a specific disease like cancer. Believes gray and yellow tubercle to be distinct; gray specific, yellow the result of inflammation. Tuberculous and rheumatic diatheses are closely allied. Believes in only one kind of tuberculous matter, viz: miliary. Tuberculosis is a constitutional disease, not depending on inflammation; scrofula is dependent on inflammation; usually attacks the glands, and is due to yellow deposit in the glands and sometimes in the lungs. It is the result of inflammation; should always push the treatment. Doctors have no right to tell any man he is going to die. Be careful in diagnosis and prognisis; always have hope. We might often cure, but let our patients die through neglect. Emaciation, one of the best evidences of tuberculosis. True tuberculosis is as sure almost to end in death as cancer. One of the first evidences of consumption is prolonged expiratory murmurs; principal treatment iodine in some form over the chest; iodine, cod-liver oil, alcohol, and nutritious diet principal internal treatment. A new issue in some other portion of the body often arrests the disease, viz: fistula in ano, hemorrhoids, etc. Cod-liver oil and alcohol are highly antagonistic to consumption. This paper was evidently studied with much care by the distinguished physician who prepared it, and a lengthy and instructive discussion followed.

Meeting adjourned.

Met again at 8½ o'clock, when a large and fashionable audience graced the occasion with their presence. The President, Dr. J. S. Weatherly, proceeded to deliver the annual address. His subject was the recent act of the Legislature making the Association the State Board of Health, and the consequent duties which this act imposed upon the Association. He elaborated the importance of the duties, and showed that it was within the power of the Association, by proper action, to greatly benefit the State by reducing, if not entirely destroying, the fatal effects of all preventable diseases, such as cholera, small pox, etc. He denominated miasma as the greatest impediment to the increase of of our population by immigration, and argued that by a proper system of drainage, etc., under the direction of the State Board of Health, much could be done towards removing this impediment. He argued that all moral diseases and bodily infirmities, which were transmitted from generation to generation, were, to to a great extent, susceptible of prevention, and that they should be brought under the control of the State through the Board of Health. His address was delivered in an easy and graceful manner, and was listened to attentively by quite a large audience of ladies and gentlemen.

---

*April* 14, 1875.—According to adjournment, the Association was called to order by the President.

An interesting and lengthy paper was read by Dr. Jerome Cochran, of Mobile, on small-pox. He said most epidemics abate and die out during the winter; but not so with small-pox. The greatest mortality is in the winter. The paper contained some valuable statistics of this disease in New Orleans and Mobile. To attempt a synopsis of the able document, we feel is an act of injustice to the writer. A discussion followed, and was listened to with marked attention.

Dr. Gaines spoke at length on the importance of vaccination. Believes the people should be vaccinated, even though some of the family already have small-pox. Many are prejudiced against vaccination during the prevalence of this disease. They believe it will contaminate them. Everybody should be vaccinated during an epidemic. Thinks many have it because they are not properly vaccinated. Related the case of a mother who took small-pox in its worst form. Six children were vaccinated on the sixth or seventh day after she was attacked, and not one of them took the disease.

Dr. Baldwin related a similar case, only the number vaccinated was twelve, and all were exempt.

Dr. Cochran believes that vaccination does not exempt for life, but we cannot tell how long it will exempt. Many have the disease who have been properly vaccinated. Does not think protection entirely complete at any time. Protection is due to the amount and character of the marks. Believes a certain per cent. of very sore arms would result, were they only scratched with a pin or any other instrument. Does not think bad sores necessarily follow vaccination. Vaccine crust is not pus, but a growth which drops off when matured. Is inclined to think that lymph used from the arm of a syphilitic patient would not transmit the disease, so long as pure lymph is used, and no pus. True vaccine crust is amber-colored and transparent. Can tell whether a crust came off a white person or negro, also a blonde or brunette, by its appearance. Humanized virus was more certain as a protection than bovine.

Discussion on this subject closed.

Dr. Bradfield read a paper on puerperal convulsions and delivery with forceps. Delivery was followed for some days by convulsions. Had happy results from hypodermic injections of morphine. Also exhibited Esmarch's apparatus for bloodless operations.

Association adjourned.

Met at 3 P.M., and, after an animated discussion on Constitution, etc., elected five new Counselors.

Adjourned to meet at 8½ o'clock, to hear the address of Dr. Ketchum.

Long before the hour of speaking arrived, the hall was packed to overflowing by an intelligent audience, and among them were many of Montgomery's fairest and most beautiful ladies.

The President introduced the orator of the evening, Dr. Geo. A. Ketchum of Mobile, who rose amidst applause. His dignity, commanding appearance and general bearing bespoke for him a man of no mediocre attainments. His address on Medical Ethics was sound and replete with wisdom, interspersed with flights of eloquence that would have done honor to some of the most distinguished orators of our country. His eulogy on the medical profession was truly sublime, and his peroration on some of its members unsurpassed for beauty of language and expression.

*April* 15, 1875.—Board of Counselors met at 9 o'clock, and proceeded to elect ten additional Counselors.

The Association came to order at 10 o'clock, at the call of the President.

The annual report of the Board of Censors, a very able paper, was read by Dr. Jerome Cochran, of Mobile.

A telegram was received from the State Medical Association of South Carolina, bearing words of greeting to the Medical Association of Alabama.

Dr. McKeithen, of Prattville, presented a paper on a remarkable case of twin gestation. A colored woman gave birth, in Prattville, last March, to two children—one a mulatto, the other a full-blooded negro. Fifteen minutes elapsed between the delivery of the two. The dark child was born first. The umbilical cords were distinct, and entered the placentæ at a common point. The placentæ were intimately blended. The mother of the children is very black, a true African, with kinky hair and flat nose. She had been marrried ten years to a husband of the same type as herself, and had borne children previously, who were all black, resembling her husband and herself. There was a marked difference in these two children. One was a bright colored mulatto, with straight, reddish, sandy-looking hair, and hazel eyes; the other was black, with flat nose, thick lips, black kinky hair and black eyes. Both the children are doing well. The bright one looks the healthier; the black one is the larger. He believes the children are the offspring of two fathers, differing in "race, color, and perhaps previous condition of servitude."

He related a second case, of a lady in good standing, who gave birth to twins, in the practice of one of his friends. The lady was married, and gave birth to twins, both white, but so unlike that it arrested the attention of the accoucheur, and he determined to investigate. As the children grew, their features became more unlike. One had black hair, dark eyes and skin—the image of the husband; the other had bright sandy hair, blue eyes and fair complexion—exactly similar to the features of a drug clerk who was intimate with the family. The doctor visited the mother, and, with threats of exposure, received a confession. She said her husband had left home, to be absent two weeks, and she received the drug clerk in her embraces. Just as he had completed the illicit intercourse, the husband unexpectedly returned. He walked in at the front door, and the clerk, unperceived, went out the back way. Coition took place at once between the husband and wife; the wife conceived and gave birth to twins—another evidence that twins can be born with different fathers. He related also a third case in favor of his position.

Dr. Riggs submitted an able paper on malaria, handling the subject in an able and scientific manner.

Dr. Gill read a short paper on lithotomy. Has operated seventeen times with success throughout. He claims priority in the median operation. He also reported the case of a spear-point being taken from the brain of a man after it had remained there two years. He turned back a flap—found the end of the spear-point on a line with the external surface of the cranium—filed the skull on either side—caught the steel with a pair of tooth forceps, and extracted it. It had been imbedded in the brain substance one inch. The man had had many convulsions during the time. No unpleasant symptoms after its removal, and a perfect recovery resulted.

Dr. Gaines made some extemporaneous remarks on diphtheria. He thinks this disease occurs in any portion of the body. Has often seen it beginning in the nose. Related several cases of interest. Has seen the whitish exudation on any portion of the body where an abrasion exists or a wound has been made. When the stomach becomes affected, patients nearly all die. The heart often ceases to act, producing death. Does not know whether pneumogastric is primarily affected or not. Dr. Gaines was requested to put his remarks in writing, for publication.

Association adjourned.

The Association met at 2½ o'clock.

Dr. Griggs, of Georgia, entertained the meeting with an interesting address, and referred to the cordial manner in which he had been received in Montgomery.

The election of officers for the ensuing year then took place, with the following result:

President, Dr. J. J. Dement, Huntsville; 1st Vice-President, Dr. P. Bryce, Tuscaloosa; 2d Vice-President, Dr. F. M. Peterson, Greensboro; Censor, Dr. W. H. Anderson, Mobile; Orator, Dr. R. F. Michel, Montgomery—alternate, Dr. C. Toxey, Mobile.

Board of Health Officers—Dr. G. A. Ketchum, five years; Dr. E. A. Semple, four years; Dr. E. P. Gaines, three years; Dr. C. D. Parke, two years; Dr. S. D. Seelye, one year.

Dr. B. H. Riggs was re-elected Secretary, and Dr. W. C. Jackson Treasurer.

Dr. Seelye, of Montgomery, offered a prize of $100 for the best essay on Bright's disease of the kidneys, to be decided by committee at next meeting—open to the world.

Dr. Ketchum offered a resolution of thanks to the several railroads for their courtesies; also to the citizens of Montgomery for their hospitalities.

The Association adjourned, to meet in Mobile on the second Tuesday in April, 1876.

During the stay of the Association in Montgomery, many receptions were given, and nothing was left undone to make its visit pleasant and attractive.

